# Micrographic Evaluation of Water-in-Oil Emulsion Stability up to Critical Electric Field

**DOI:** 10.1021/acsomega.6c02577

**Published:** 2026-10-01

**Authors:** Troner Assenheimer, Hyago Braga dos Santos, Thiago Americano do Brasil, Edson Watanabe, Frederico Wanderley Tavares, Márcio Nele

**Affiliations:** † Laboratory for Applications in Petroleum and Biofuels (LABAP), Graduate Program in Mechanical Engineering (PGMEC), 28110 Federal Fluminense University, 24210-240 Niterói, Brazil; ‡ Electrical Engineering Program COPPE, Federal University of Rio de Janeiro, 21941-972 Rio de Janeiro, Brazil; § Celso Suckow da Fonseca Federal Center for Technological Education − CEFET, 20271-110 Rio de Janeiro, Brazil; ∥ Chemical Engineering Program COPPE, Federal University of Rio de Janeiro, 21941-972 Rio de Janeiro, Brazil

## Abstract

Electrostatic demulsification is widely applied in the petroleum industry for the separation of water-in-oil emulsions; however, the relationship between microscopic droplet dynamics and the macroscopic electrical response remains insufficiently understood, particularly regarding the definition of the critical electric field (CEF). This work presents a quantitative investigation of electrocoalescence using optical microscopy coupled with electrical current monitoring under a direct current electric field. Water-in-oil (W/O) model emulsions were evaluated through a factorial experimental design comprising 81 experiments, in which the electrode gap (100–300 μm), water cut (5–15 wt %), and surfactant concentration (0.5–1.5 wt %) were systematically varied. Real-time observations enabled the identification of droplet polarization, chain formation, coalescence, and the development of conductive pathways. The CEF, ranging approximately from 0.7 to 4.0 kV/cm, was determined from the simultaneous occurrence of current spikes and the visual formation of coalesced droplets that establish conductive pathways within the emulsion. The results demonstrate that droplet alignment occurs at the initial stages of electric field application and does not correspond to a significant increase in current. Instead, the abrupt rise in current was associated with the growth of droplets that establish electrical conduction, indicating that the CEF corresponds to the final stage of the electrocoalescence process. Statistical analysis revealed a pronounced quadratic effect of water cut, while electrode spacing and surfactant concentration also exerted significant influences through linear, quadratic, and interaction effects. These findings provide mechanistic insight into electrocoalescence and support a revised interpretation of the critical electric field, contributing to the understanding and potential optimization of electrostatic separation processes.

## Graphical abstract

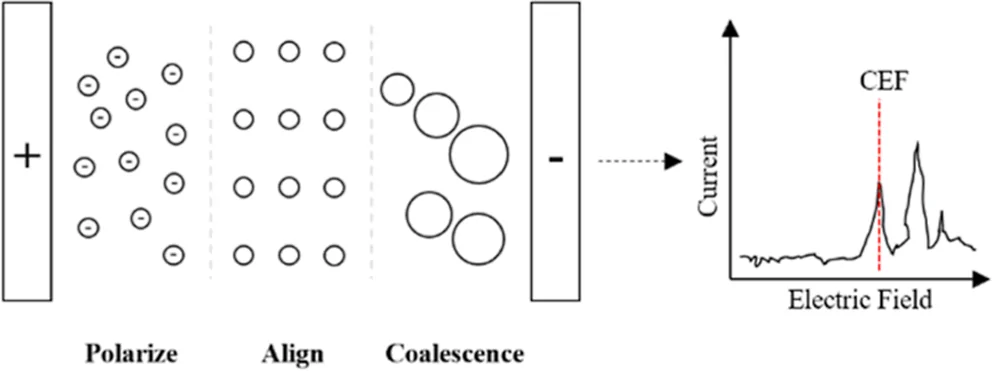

## 1 Introduction

Crude oil production streams inherently contain water, either as residual formation water or as water injected during secondary and tertiary recovery operations to maintain reservoir pressure and enhance oil displacement efficiency.^1,2^ The coexistence of hydrocarbons, brine, and naturally occurring surface-active species promotes the formation of water-in-oil (W/O) emulsions with varying stability characteristics.^3,4^

Prior to transportation and refining, the water content is required to be reduced in order to mitigate corrosion, prevent catalyst deactivation, minimize fouling, and ensure reliable operation of downstream processing units.^5^ Separation of the dispersed aqueous phase is achieved through gravitational settling,^6,7^ chemical demulsification,^8^ thermal treatment,^9^ and electrostatic methods.^10,11^ Among these technologies, electrostatic demulsification has become widely adopted in industrial practice due to its high energy efficiency, reduced chemical demand, and comparatively lower environmental impact.^12^

Modern inline electrocoalescers operate in continuous flow and promote droplet growth by polarizing dispersed water droplets, thereby enhancing dipole-driven interactions, collision frequency, and coalescence under applied electric fields.^13–15^ The resulting enhancement in droplet diameter reduces sedimentation time according to hydrodynamic principles consistent with Stokes’ law.^16^ Despite decades of industrial use, the optimization of electrocoalescence performance remains constrained by incomplete understanding of the coupled electrohydrodynamic, interfacial, and physicochemical mechanisms governing droplet deformation, film drainage, and coalescence under electric forcing.^17^

Early observations by Pearce^18^ documented droplet chain formation through photographic techniques following the application of electric fields. Chen et al.^19^ combined experimental measurements with numerical simulations to demonstrate that electrostatic interactions drive droplet alignment and chaining in W/O emulsions. Luo et al.^20^ investigated dipole–dipole interactions and dielectrophoretic effects associated with nonuniform electric fields, highlighting their influence on droplet aggregation dynamics. However, these investigations primarily provided qualitative insights, and quantitative relationships between electric field intensity, microstructural evolution, and macroscopic electrical response remain limited.

The stability of water-in-oil emulsions and their response to externally applied electric fields are strongly governed by interfacial composition and physicochemical interactions.^21,22^ Naturally occurring surface-active species, including asphaltenes, resins, and fine solids, form viscoelastic interfacial films that impede droplet approach and retard film drainage, thereby increasing resistance to coalescence.^21^ The presence of polymers, surfactants, and dissolved salts modifies interfacial tension, electrical properties, and droplet mobility, leading to complex stabilization mechanisms associated with steric hindrance and electrostatic interactions.^21–23^ Experimental observations^21^ and molecular-scale analyses^22,23^ demonstrate that increasing surfactant concentration enhances interfacial rigidity and suppresses droplet merging, whereas variations in ionic strength and nanoparticle content alter charge distribution and interfacial structure, thereby influencing electrocoalescence kinetics and separation efficiency.

From a mechanistic standpoint, electrocoalescence has been interpreted within the framework of electrohydrodynamics,^24^ in which droplet polarization induced by an electric field generates dipole–dipole attractions that promote alignment, deformation, and eventual coalescence. Detailed experimental^25^ and numerical^24^ investigations identify multiple coalescence regimes, including complete coalescence, partial coalescence, and stretching behavior, governed by the interplay among electric field intensity, droplet size, fluid viscosity, and interfacial properties. Numerical simulations employing sharp-interface formulations and phase-field approaches provide quantitative descriptions of liquid bridge formation,^24^ neck evolution, and regime transitions, revealing the existence of threshold conditions that delineate distinct coalescence outcomes, emphasizing the inherent complexity associated with predicting electrocoalescence behavior in realistic crude oil emulsions.

The characteristics of the applied electric field, including waveform,^26^ frequency,^27^ and spatial distribution,^28^ exert a decisive influence on electrostatic separation performance. Comparative analyses of direct current, alternating current, and pulsed electric fields reveal significant differences in droplet deformation dynamics, threshold field intensity, and separation efficiency.^27^ Pulsed and modulated electric fields introduce frequency-dependent effects that modify droplet response and alter the onset of coalescence,^29^ whereas nonuniform electric fields generate dielectrophoretic forces that drive droplet migration toward regions of elevated field strength. Furthermore, advances in electrode design, such as matrix electrode configurations,^28^ and the integration of coupled electric-magnetic fields demonstrate enhanced dehydration efficiency and reduced energy consumption, indicating promising opportunities for process intensification and improved operational performance.

Recent developments emphasize the integration of advanced modeling strategies^30^ and data-driven methodologies^31^ to improve predictive understanding of electrocoalescence phenomena. Machine learning frameworks trained on experimental data sets^31^ have been employed to quantify the influence of operating variables, including applied voltage, frequency, electrode spacing, and droplet population characteristics, on coalescence behavior and droplet size distribution. Complementary molecular dynamics investigations^23,30,32^ provide atomistic insight into charge redistribution, surfactant rearrangement, and nanoscale interfacial processes under electric forcing. The convergence of multiscale modeling and experimental diagnostics represents a significant step toward the development of predictive tools capable of supporting the optimization, scale-up, and digitalization of electrostatic separation technologies.

The critical electric field (CEF) concept has been introduced as a quantitative descriptor of emulsion stability, representing the threshold electric field required to induce rapid conductivity changes associated with droplet bridging or coalescence events.^33^ This approach has been employed in several studies to compare stability across crude systems and operating conditions.^33–38^ These technologies demonstrated that the application of direct current between electrodes leads to droplet polarization, alignment along the electric field direction, and the formation of conductive pathways, manifested as a sudden increase in measured current. Within this framework, the CEF corresponds to the electric field intensity at which this abrupt transition occurs.

From an industrial perspective, inadequate control of electrocoalescence conditions often leads to reduced separation efficiency,^14^ increased chemical consumption,^39^ higher energy demand,^12^ and operational instability in large-scale separators.^40^ Variability in crude composition and operating conditions further complicates the translation of laboratory observations to field applications.^40^ Therefore, establishing robust correlations between operating variables, microstructural behavior, and electrical response is essential for improving separator design, enhancing operational reliability, and supporting the digitalization and optimization of production facilities.

However, a critical limitation in the current literature lies in the absence of integrated methodologies that simultaneously capture electrical signatures and direct microstructural evolution during electrocoalescence. Most studies rely exclusively on conductivity or current measurements without visual confirmation of droplet dynamics, thereby limiting mechanistic interpretation of the onset of conductive pathways. In addition, systematic investigations that address the combined effects of geometric parameters, such as electrode spacing, and formulation variables, including water cut and surfactant concentration, remain scarce. This lack of quantitative and mechanistically grounded understanding hinders the development of predictive tools required for process optimization and scale-up of electrostatic separation units.

In the present study, the stability of water-in-oil emulsions is investigated through simultaneous measurement of the critical electric field and real-time optical microscopy, enabling direct correlation between electrical behavior and droplet-scale phenomena during electrocoalescence. Continuous monitoring of electric current under controlled electric field application, combined with time-resolved micrographic analysis, provides a reproducible methodology for identifying the onset of conductive transitions and coalescence events. The objective of this work is to elucidate the mechanisms governing emulsion destabilization under electric fields and to establish a quantitative framework for CEF determination supported by direct visual evidence.

An experimental apparatus was designed and constructed to systematically evaluate the influence of electrode gap (GP), water cut (WC), and surfactant concentration (SC) on droplet interactions, conductive network formation, and emulsion stability. By integrating electrical diagnostics with microscopic observation, this study provides insights into the fundamental mechanisms controlling electrocoalescence and offers guidance for the optimization and scale-up of electrostatic separation processes in petroleum production systems.

## 2 Methodology

Prior to the development of the proposed experiments, the water-in-oil emulsions were prepared using the synthetic oil Exxol D130 as the continuous phase and deionized water (electrical conductivity of 1.14 μS·cm^–1^ at room temperature) as the dispersed phase. Emulsion stabilization was achieved through the addition of two surfactants: Span 80 (sorbitan monooleate, nonionic surfactant, molecular weight of 428.61 g·mol^–1^, Tedia) and AOT (sodium dioctyl sulfosuccinate, anionic surfactant, molecular weight of 444.59 g·mol^–1^, Sigma-Aldrich). A surfactant mixture composed of Span 80 and AOT at a mass ratio of 9:1 was dissolved in the oil phase under stirring until complete dissolution prior to emulsification.^41^ The selected blend was employed to achieve a hydrophilic–lipophilic balance suitable for stabilizing water-in-oil systems, considering that the hydrophilic–lipophilic balance values of Span 80 and AOT are 4.3 and 32, respectively.^42^

Emulsification was carried out using a high-shear homogenizer (Ultra Turrax, IKA T25) equipped with an S18N-19G rotor-stator assembly with a nominal diameter of 12.7 mm. The mixture was processed at a rotational speed of 3400 rpm for 3 min to produce a total emulsion volume of 100 mL. The homogenizer was operated under identical conditions for all preparations to ensure reproducibility of the droplet size distribution.

All emulsions were prepared at ambient laboratory conditions and were used shortly after preparation to minimize aging effects, such as droplet coalescence or Ostwald ripening (physicochemical destabilization). The preparation protocol was maintained consistent throughout the experimental campaign in order to isolate the effects of the investigated variables. The physicochemical properties of the fluids employed in this study are summarized in Table 1.

**Table 1:** Properties of the Considered Fluids

variable	system	units	values
interfacial tension	exxsol + deionized water	mN/m	42.5 ± 1.3
	exxol (0.5 wt % Span 80 and AOT) + deionized water		5.3 ± 0.2
conductivity	exxsol	nS/m	0.01 ± 0.001
dynamic viscosity	exxsol	mPa·s	3.2 ± 0.01
	deionized water		0.89 ± 0.01
density	exxsol	g/cm^3^	0.81 ± 0.01
	deionized water		0.99 ± 0.01

### 2.1 Characterization of Emulsions and Fluids

The stability of the emulsions under gravitational conditions was evaluated using a multiple light scattering technique (Turbiscan LAB Expert, Formulaction). This instrument measures the intensity of transmitted and backscattered near-infrared light as a function of sample height, which is directly related to the local concentration and size distribution of dispersed water droplets within the sample. Measurements were conducted in cylindrical glass cuvettes, and the samples were scanned along the vertical axis at predefined time intervals to monitor temporal changes in the dispersion state.

The instrument output is reported as profiles of transmitted and backscattered light flux expressed as a percentage as a function of sample height (mm).^43^ In the present study, only backscattered light profiles were analyzed, since all emulsions exhibited negligible light transmission due to their opacity. Variations in backscattering intensity were interpreted as indicators of droplet migration, creaming, and coalescence phenomena occurring during gravitational separation. All measurements were performed at 25 °C under controlled laboratory conditions.

Droplet size determination was performed by optical microscopy. Emulsion micrographs were acquired using an inverted optical microscope (Carl Zeiss Axiovert 40 MAT) equipped with a digital camera (Axiocam MRc, 1.4 megapixels) and image acquisition software (Axiovision, version 4.8.1). Observations were conducted in bright-field mode with transmitted light, without optical filters, in order to avoid modification of light properties during image acquisition. Imaging conditions, including magnification, illumination intensity, and exposure time, were maintained constant for all samples to ensure comparability.

Droplet size analysis was carried out using *ImageJ* software.^44,45^ Images were processed using a consistent thresholding procedure applied uniformly to all samples to convert grayscale images into binary format for droplet identification. The same segmentation parameters were maintained throughout the analysis in order to minimize operator-dependent variability. When manual corrections were required to separate overlapping droplets or exclude artifacts, predefined selection criteria were applied systematically. The equivalent circular diameter was calculated for each identified droplet. For each emulsion, at least 200 individual droplets were measured to determine the average droplet diameter. Measurements were performed on three independently prepared samples, and the reported values correspond to the mean of replicate analyses to ensure statistical reliability and to reduce the influence of operator bias.

### 2.2 Electric Field

The electrocoalescence cell employed for determination of the critical electric field consisted of an acrylic housing containing a central cylindrical cavity with a diameter of 10 mm, bounded on both sides by parallel brass electrodes. The separation distance between the electrodes was adjustable within the range of 100 to 300 μm, allowing precise control of the electric field strength for a given applied voltage. The electrode assembly was mounted on the stage of an optical microscope to enable simultaneous visualization of droplet behavior during electric field application.

Electrical connections were established between the electrodes and a direct current power supply (Agilent Model 6634B) using shielded cables to ensure stable voltage delivery. The applied voltage was controlled through the power supply, and the corresponding electric field was determined based on the electrode spacing. A schematic representation of the experimental setup is shown in Figure 1.

**Figure 1:**
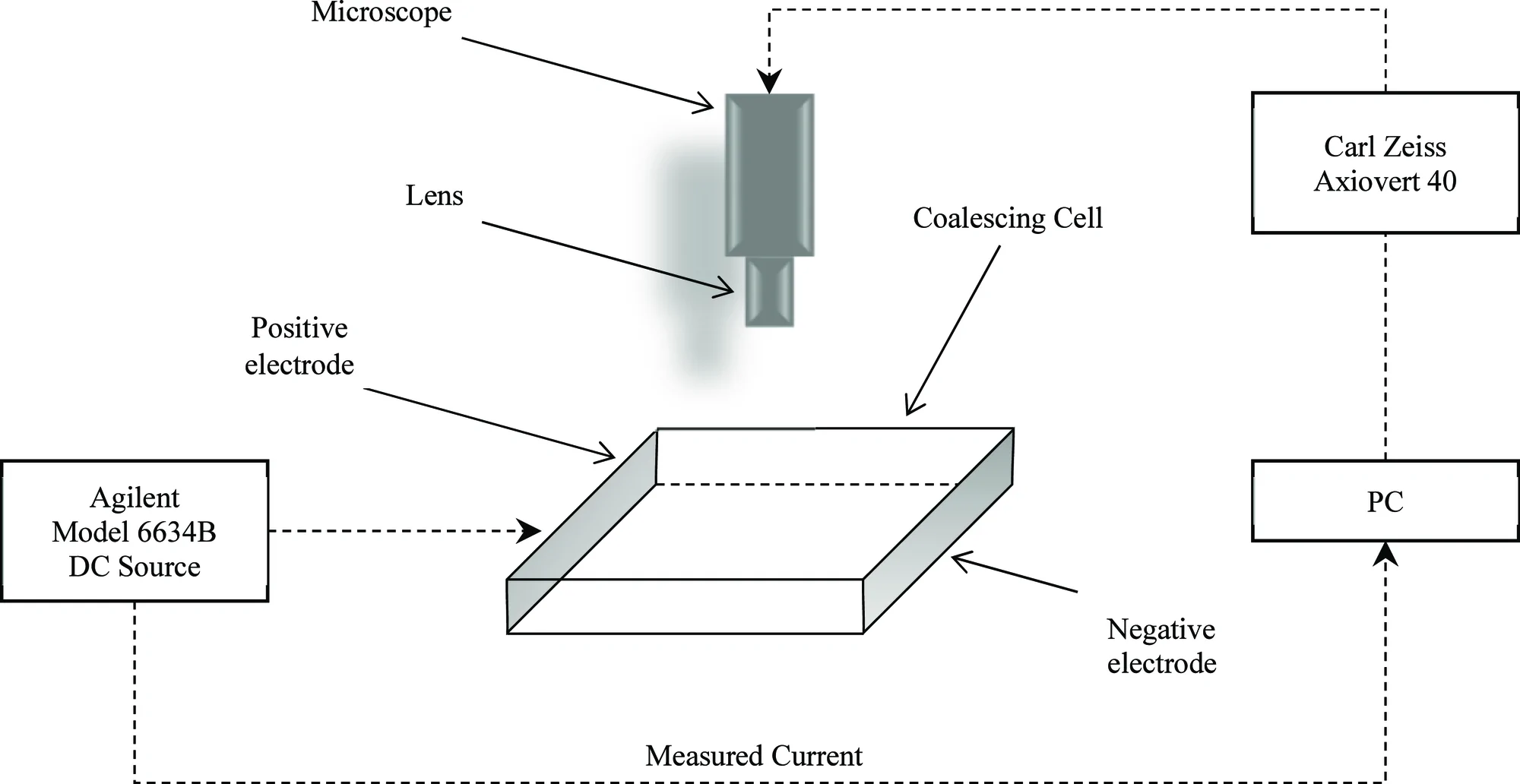
Experimental setup of the coalescing unit.

Prior to each measurement, the emulsion sample was carefully introduced into the gap between the electrodes using a microsyringe in order to avoid the entrapment of air bubbles. The electric field across the electrode plates (Figure 2) was increased linearly at a rate of 0.1 V·s^–1^ until the critical electric field condition was reached. This ramp rate was selected to provide quasi-steady conditions, allowing sufficient time for droplet polarization, deformation, and interaction processes to develop while minimizing transient overshoot and electrical noise that could affect the accurate identification of the onset of conductive pathways. All experiments were conducted at ambient laboratory temperature, and identical procedures were followed for all measurements to ensure reproducibility.

**Figure 2:**
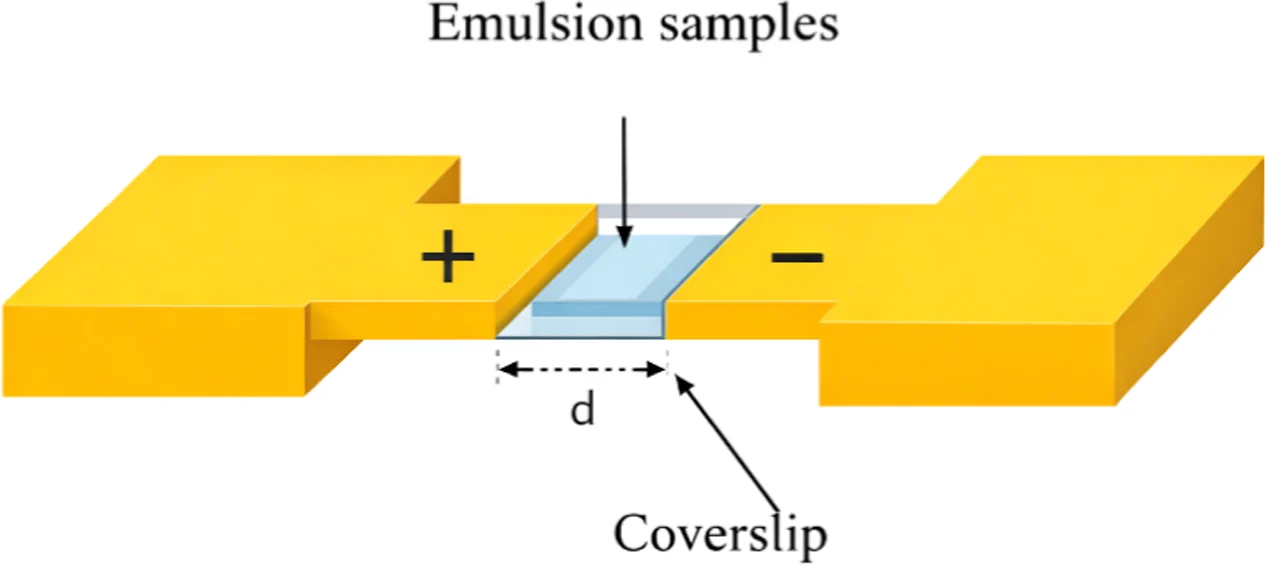
Electrodes plates setup.

### 2.3 Experimental Design

A systematic experimental design was developed to investigate the effects of key operational and formulation variables on the critical electric field associated with electrocoalescence. The selection of factors was based on their known influence on droplet interactions, polarization behavior, and conductive pathway formation within water-in-oil emulsions. The independent variables considered in this study consisted of electrode spacing, water cut, and surfactant concentration.

Electrode spacing was varied at three levels (100, 200, and 300 μm) in order to evaluate the influence of electric field distribution and geometric confinement on droplet alignment and coalescence dynamics. Water cut was investigated at 5, 10, and 15 wt % to assess the effect of dispersed phase concentration on droplet collision frequency and electrical conductivity of the emulsion. Surfactant concentration was varied at 0.5, 1.0, and 1.5 wt % to examine its role in interfacial film strength, emulsion stability, and resistance to coalescence. The response variable corresponded to the measured value of the critical electric field obtained under each experimental condition.

The experimental matrix followed a full factorial design with three factors at three levels (3^3^), enabling evaluation of both main effects and interaction effects among variables. Two independent replicates were conducted at each experimental condition using separately prepared emulsions to ensure statistical reliability and to account for experimental variability associated with sample preparation and measurement procedures. This approach resulted in a total of 81 experimental runs, which were subsequently analyzed using *Statistica* software.

All experiments were performed following identical preparation and measurement protocols in order to ensure consistency across the data set. The complete experimental design and factor combinations are presented in Table 2.

**Table 2:** Complete Experimental Design

experiment	GP (μm)	WC (wt %)	SC (wt %)
1	100	5	0.5
2	100	5	1.0
3	100	5	1.5
4	100	10	0.5
5	100	10	1.0
6	100	10	1.5
7	100	15	0.5
8	100	15	1.0
9	100	15	1.5
10	200	5	0.5
11	200	5	1.0
12	200	5	1.5
13	200	10	0.5
14	200	10	1.0
15	200	10	1.5
16	200	15	0.5
17	200	15	1.0
18	200	15	1.5
19	300	5	0.5
20	300	5	1.0
21	300	5	1.5
22	300	10	0.5
23	300	10	1.0
24	300	10	1.5
25	300	15	0.5
26	300	15	1.0
27	300	15	1.5

## 3 Results and Discussions

Considering the proposed methodology and the experimental configuration employed in this study, the emulsions were prepared under controlled conditions and their microstructural characteristics were evaluated in order to obtain a detailed understanding of their initial state prior to electrocoalescence experiments. The assessment of emulsion morphology was performed to verify the consistency of the preparation procedure, ensuring that variations in subsequent measurements were primarily associated with the investigated variables rather than differences in droplet structure.

Figure 3 and Table 3 present representative micrographs and the corresponding quantitative characterization of the emulsions prepared for this work. Visual inspection of the micrographs indicates a slight apparent decrease in droplet size with increasing surfactant concentration, which is consistent with the expected enhancement of interfacial stabilization and increased resistance to droplet coalescence during emulsification. However, quantitative analysis revealed that the observed variations in average droplet diameter remained within the experimental uncertainty associated with the measurement procedure.

**Figure 3:**
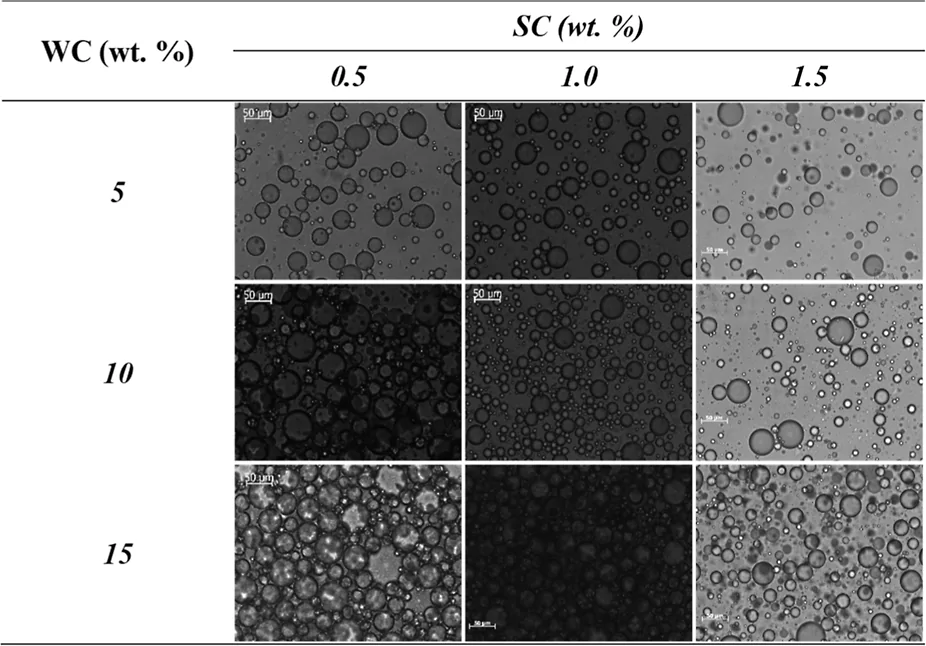
Micrograph of the emulsions used in the experiments.

**Table 3:** Average Diameter of Droplets in Micrometers

	droplet diameter (μm)		
WC (wt %)	0.5 wt % SC	1 wt % SC	1.5 wt % SC
5	20.0 ± 1.0	19.4 ± 1.0	16.6 ± 0.8
10	17.4 ± 0.9	15.8 ± 0.8	15.1 ± 0.8
15	13.7 ± 0.7	12.9 ± 0.6	12.1 ± 0.6

Considering the results presented in Figure 3 and in Table 3, it was possible to observe that the emulsions exhibited comparable droplet size distributions across the tested conditions, suggesting that the addition of surfactant within the investigated range did not produce statistically significant changes in the initial morphology. Consequently, the emulsions were considered morphologically similar, which supports the assumption that differences observed in electrocoalescence behavior arise primarily from interfacial and electrohydrodynamic effects rather than from initial droplet size variations.

To evaluate emulsion stability under gravitational conditions, sedimentation experiments were conducted using multiple light scattering analysis in order to distinguish stability differences among the prepared emulsions. The temporal evolution of the dispersion state was monitored through laser light profiling, which provides spatially resolved information on droplet migration, concentration gradients, and phase separation phenomena.

Figure 4 presents the stability profiles for all investigated water cuts and surfactant concentrations obtained 30 min after emulsion preparation. A gradual sedimentation process was observed for all experimental conditions, as evidenced by an increase in backscattered light intensity near the bottom region of the sample vials (left side of the profiles) accompanied by a corresponding decrease in backscattering at the upper region. These trends indicate the downward migration of dispersed water droplets driven by density differences between the dispersed and continuous phases, leading to the formation of a sedimented layer over time.

**Figure 4:**
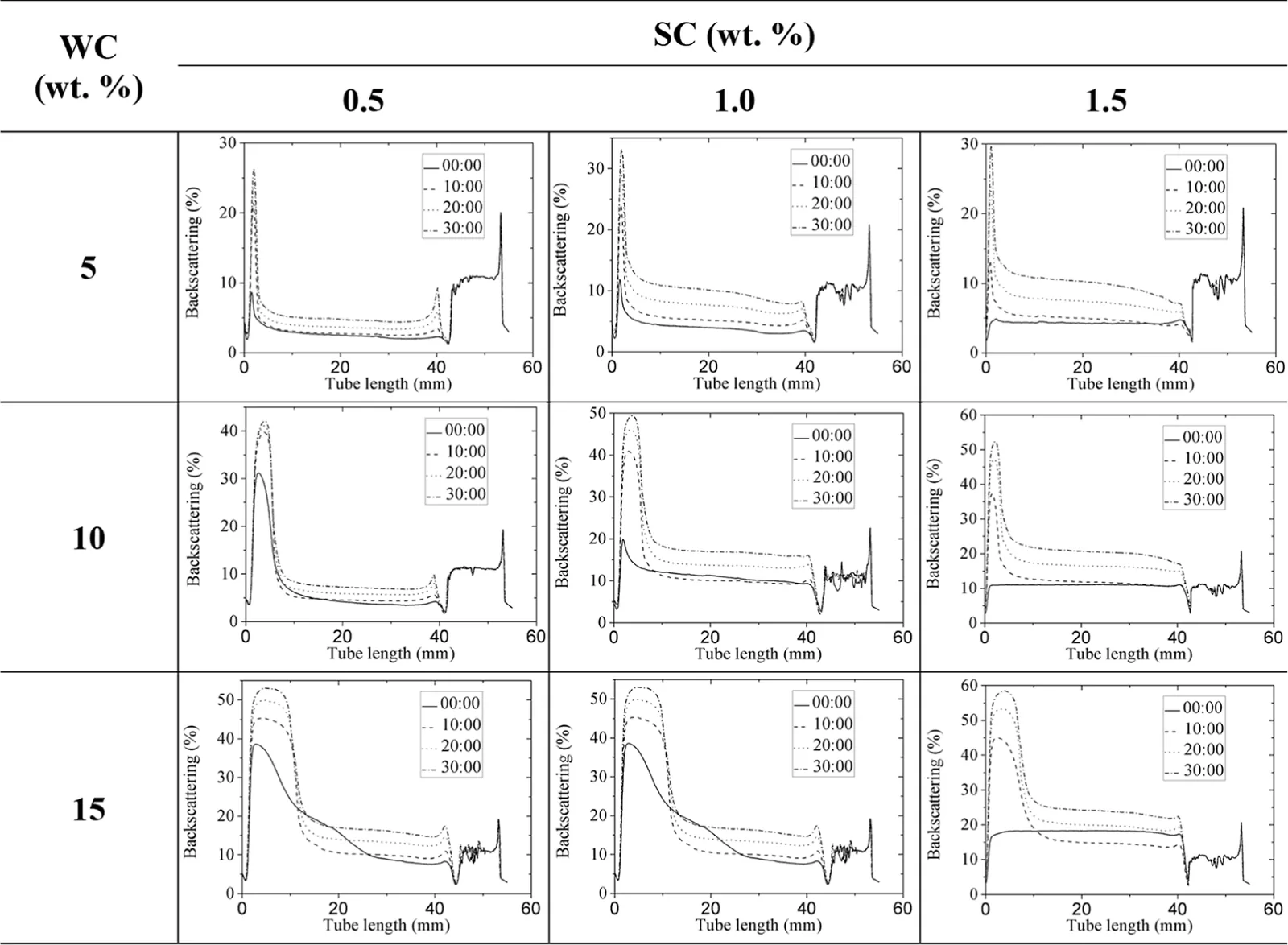
Results for laser light scattering profiling as a function of tube length for the time interval up to 30 min after emulsion preparation.

The occurrence of sedimentation is consistent with the relatively large droplet diameters measured in this study, which favor gravitational settling when hydrodynamic drag is insufficient to maintain dispersion stability. The dynamic viscosity of Exxol D130, reported in Table 1, suggests limited resistance to droplet motion, thereby facilitating sedimentation under quiescent conditions.

These observations are in agreement with previous studies by França et al.^46^ and Balsamo et al.,^47^ who reported comparable backscattering profiles and sedimentation kinetics for water-in-oil emulsions with similar droplet size ranges and continuous phase viscosities. In particular, those studies highlighted that gravitational separation becomes significant when droplet sizes exceed the threshold at which Brownian motion no longer dominates transport processes, which is consistent with the behavior observed in this work.

From a critical perspective, the absence of pronounced differences among stability profiles across the investigated conditions indicates that variations in surfactant concentration and water cut within the studied range did not substantially alter the gravitational stability on the time scale considered. This suggests that interfacial films formed by the Span 80/AOT mixture provided comparable resistance to droplet aggregation under static conditions, and that droplet size distribution remained the dominant factor governing sedimentation behavior. Such findings reinforce the interpretation that the emulsions prepared in this work exhibit similar baseline stability, which is essential for isolating the effects of electric field application in subsequent experiments.

The implications of these results for electrocoalescence are significant. Since gravitational destabilization proceeds slowly and uniformly across conditions, the observed differences in critical electric field are unlikely to originate from pre-existing macroscopic separation or large variations in droplet distribution. Instead, the electrocoalescence response is expected to be governed primarily by electrohydrodynamic interactions, droplet polarization, and interfacial film properties under applied electric fields. Establishing this baseline stability ensures that the electrocoalescence experiments reflect intrinsic electrical effects rather than artifacts associated with uncontrolled sedimentation or heterogeneity in the initial emulsion structure. Finally, droplet size estimates derived from analysis of the backscattering profiles were consistent with the average diameters obtained from optical micrographs (Figure 3), further confirming the reliability of the characterization methods and supporting the conclusion that the emulsions were comparable prior to electrical testing.

### 3.1 Stability under Electric Field

As described in Section 2.2, the electrocoalescence experiments were conducted using the custom cell coupled to an optical microscope, which enabled direct visualization of droplet dynamics under applied electric fields across the full factorial experimental design (3^3^, Table 2). This configuration allowed simultaneous monitoring of microstructural evolution and electrical response for all 81 experimental conditions investigated in this study.

For the range of operating conditions explored and considering the geometry of the experimental apparatus, the characteristic behavior associated with the critical electric field did not follow the description reported by Mhatre et al.,^48^ in which elongated droplets bridge the electrode gap and establish a continuous conductive pathway. In the present experiments, merged droplets did not extend toward the electrodes sufficiently to form stable bridges that spanned the gap. Instead, the electrocoalescence process exhibited distinct morphological features that were consistent across all conditions.

Figure 5, corresponding to experiment 21, provides a representative sequence illustrating the general behavior observed throughout the experimental campaign. The observed electrocoalescence dynamics are in agreement with the mechanisms described by Eow and Ghadiri,^49^ wherein droplet chains form through the collective motion and polarization of individual droplets under the influence of the electric field. These chains align along the direction of maximum field strength, and they do not necessarily originate or terminate at the electrodes, nor do they require direct contact with electrode surfaces.

**Figure 5:**
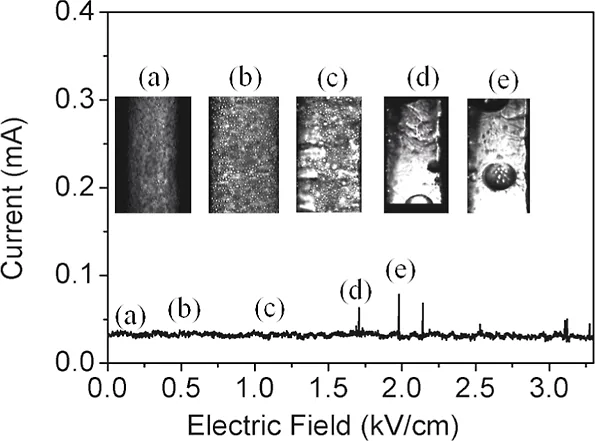
Effect of the electric field on current between the electrodes and the pictures of the electrocoalescence process. (a) Direct current electric field was initiated; (b) droplet polarization and dipole-driven alignment; (c) coalescence-driven aggregate formation; (d) quasi-stationary coalescence; (e) post-CEF intermittent current spikes and oscillations.

At the instant identified as (a), the direct current electric field was initiated. As the applied voltage increased linearly, droplets became polarized and progressively aligned with the electric field direction due to dipole–dipole interactions, as illustrated in (b). Continued field application promoted droplet approach and merging events, leading to the formation of larger aggregates through coalescence, as shown in (c). In stage (d), the coalescence process appeared to reach a quasi-stationary condition, accompanied by a marked increase in measured current, indicating the formation of transient conductive pathways within the emulsion. The electric field corresponding to this condition was defined as the critical electric field for all experiments reported in this work.

It is important to emphasize that, in contrast to several previous studies that define the critical electric field as the onset of electrocoalescence or the initial increase in current,^37,43,50–57^ the present work adopts a definition associated with the completion of the dominant coalescence events and the establishment of conductive pathways. This distinction arises from the direct visual evidence provided by microscopy, which revealed that significant droplet restructuring precedes the abrupt electrical response.

Finally, stage (e) illustrates intermittent current spikes and oscillations observed after the critical condition was reached. These fluctuations are attributed to transient formation and disruption of conductive paths, as well as to local electric field heterogeneities associated with microscopic roughness of the electrode surfaces, consistent with interpretations reported in the literature.^48,58–60^

The criterion adopted for determination of the critical electric field (CEF) in the present experiments was based on the combined analysis of electrical measurements and direct visual observations. Specifically, two conditions were considered simultaneously: the occurrence of the first significant spike in the measured current and the visual confirmation that droplet growth had progressed to a stage at which sufficiently large droplets or aggregates were capable of establishing conductive pathways within the emulsion. This combined approach ensured that the identified CEF corresponded to a physically meaningful transition associated with the development of current flow through the dispersed system.

Figure 6 presents representative results for experiments 3 (a), 9 (b), 12 (c), 18 (d), 21 (e), and 27 (f), corresponding to the conditions listed in Table 2. These experiments were conducted at the highest surfactant concentration investigated (1.5 wt %) and at water cuts of 5 and 15 wt %, with electrode gaps of 100, 200, and 300 μm. The remaining experiments exhibited qualitatively similar behavior.

**Figure 6:**
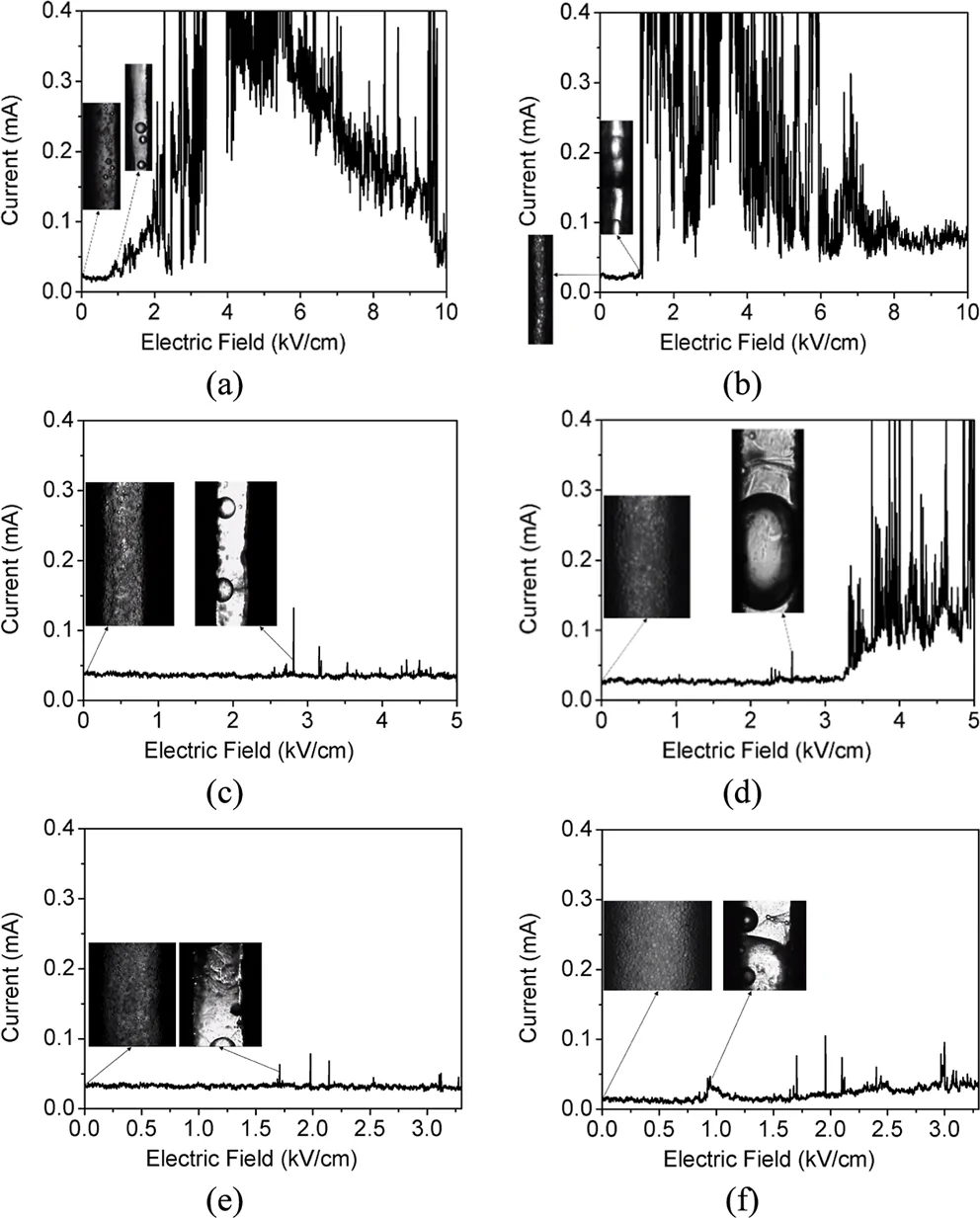
Current vs Electric field plot for the experimental conditions 3 (a), 9 (b), 12 (c), 18 (d), 21 (e) and 27 (f).

An important observation concerns the influence of electrode spacing on the electrocoalescence response. Increasing the gap between electrodes reduced the electric field intensity for a given applied voltage, which consequently required higher voltages to achieve comparable polarization conditions and led to higher apparent CEF values. From an energetic perspective, this behavior reflects a reduction in energy density delivered to the emulsion, consistent with observations reported in previous studies.^10,50,53,61,62^ In addition, the magnitude of the measured current decreased as the electrode separation increased, indicating reduced probability of conductive pathway formation across larger distances.

For experiments conducted at a water cut of 5 wt % (Figure 6 (a), (c), and (e)), the increase in current was observed only after extensive droplet coalescence had occurred, resulting in the formation of large droplets capable of facilitating current flow. Under these dilute conditions, droplet interactions were limited by larger interdroplet distances, requiring significant coalescence before conductive pathways could develop.

In contrast, at a water cut of 15 wt % (Figure 6 (b), (d), and (f)), current increases were associated with the presence of large droplets whose dimensions approached the electrode gap, enabling direct or near-direct connections between electrodes. Similar behavior was observed for intermediate water cuts of 10 wt %. Moreover, larger current spikes were consistently observed at higher water cuts, reflecting increased conductivity arising from shorter interdroplet distances and greater probability of droplet bridging.

Given the carried-out analyses, CEF was determined considering a combined criterion based on synchronized current measurements and optical microscopy. The CEF was assigned to the electric field corresponding to the first significant current spike, provided that this electrical event coincided with the visual evidence that the dominant coalescence process had reached its final stage. This visual condition was characterized by the formation of large coalesced droplets capable of establishing transient conductive pathways within the emulsion.

The experimentally determined CEF values are summarized in Table 4, which also reports the corresponding mean values and standard deviations for each condition, providing an assessment of measurement repeatability.

**Table 4:** Experimental Design Results for the Critical Electric Field

	CEF (kV/cm)				
experiment	replica 1	replica 2	replica 3	average CEF (kV/cm)	standard deviation
1	2.7	4.3	3.6	3.5	0.6
2	3.7	2.8	3.5	3.3	0.4
3	2.8	2.2	2.2	2.4	0.3
4	3.3	3.3	3.1	3.2	0.1
5	0.5	0.9	0.9	0.8	0.2
6	1.7	0.8	1.1	1.2	0.4
7	0.6	0.7	0.7	0.7	0.1
8	2.8	3.1	3.9	3.3	0.5
9	1.1	1.7	1.2	1.3	0.2
10	3.8	4.0	3.3	3.7	0.3
11	2.4	1.7	2.1	2.1	0.3
12	2.4	3.1	2.8	2.8	0.3
13	2.6	3.2	2.4	2.8	0.3
14	0.7	0.8	1.4	1.0	0.3
15	3.6	1.6	2.0	2.4	0.9
16	4.2	4.0	3.7	4.0	0.2
17	2.9	2.6	3.0	2.8	0.2
18	2.6	2.9	3.0	2.8	0.2
19	1.8	1.5	1.7	1.7	0.1
20	2.6	2.9	2.4	2.6	0.2
21	1.7	2.2	1.9	1.9	0.2
22	1.0	1.2	1.0	1.1	0.1
23	0.9	0.7	0.7	0.8	0.1
24	0.9	0.9	1.0	0.9	0.0
25	1.7	2.1	1.7	1.9	0.2
26	1.9	2.0	1.9	1.9	0.0
27	0.9	1.3	1.0	1.1	0.2

To quantitatively asses the effects associated with the experimental variables, including water cut (WC), surfactant concentration (SC), and gap between electrodes (GP), a quadratic response model was adopted, as presented in eq 1, which is consistent with the structure of the three-level factorial design:

1 $$y_{e}=a_{0}+\sum_{i=1}^{n}a_{i}x_{i}+\sum_{i=1}^{n}\sum_{j=1}^{n}a_{ij}x_{ij}+\sum_{i=1}^{n}a_{i}x_{i}^{2}+\varepsilon_{i}$$

where *a*_0_ represents an independent constant, *x*_*i*_ denotes the independent variables, *x*_*ij*_ corresponds to the combination of independent variables, *a*_*i*_ represents the calculated coefficient, *a*_*ij*_ is the combination of effects, and ε_*i*_ corresponds to the prediction error.

In this regard, considering the statistical approach adopted in this study and the need to assess the significance of the regression terms included in the preliminary model formulation, Table 5 presents the ANOVA results obtained for the initially evaluated model, where the letters L and Q were used to denote the linear and quadratic effects, respectively.

**Table 5:** ANOVA Results for the Initial Assessment^a^

variable	SS	df	MS	*F*	*p*
(1) gap between the electrodes (GP) (L + Q)	2.17	2	1.09	7.45	0.001
(2) water cut (WC) (L + Q)	15.90	2	7.95	54.53	0.000
(3) surfactant concentration (SC) (L + Q)	18.48	2	9.24	63.38	0.000
1·2	6.81	4	1.70	11.68	0.000
1·3	9.97	4	2.49	17.10	0.000
2·3	10.52	4	2.63	18.03	0.000
lack of fit	12.56	8	1.57	10.77	0.000
pure error	7.87	54	0.15		
total SS	88.00	80			

a In addition to these results, the regression coefficients and the p-values obtained from analysis of standardized independent variables ranging from −1 to +1, with statistically significant terms identified based on a 95% confidence level, are presented in Table 6. For the initial results, the overparametrized model achieved a coefficient of determination (*R* ^2^) of 0.7677, indicating that approximately 77% of the variability in the response was explained.

**Table 6:** Coefficients of the Multiple Linear Regression for the CEF Experiments^a^

variable	coefficient of regression	*p* value
intercept	0.64 ± 0.18	0.001
(1) gap between the electrodes (GP) (L)	–0.42 ± 0.12	0.001
gap between the electrodes (GP) (Q)	0.29 ± 0.20	0.160
(2) water cut (WC) (L)	0.42 ± 0.12	0.001
water cut (WC) (Q)	1.97 ± 0.20	0.000
(3) surfactant concentration (SC) (L)	–0.51 ± 0.12	0.000
surfactant concentration (SC) (Q)	2.09 ± 0.20	0.000
1L by 2L	0.22 ± 0.06	0.001
**1L by 2Q**	0.12 ± 0.11	**0.295**
1Q by 2L	–0.63 ± 0.11	0.000
**1Q by 2Q**	–0.19 ± 0.19	**0.327**
1L by 3L	0.16 ± 0.06	0.018
**1L by 3Q**	0.02 ± 0.11	**0.880**
**1Q by 3L**	0.14 ± 0.11	**0.213**
1Q by 3Q	–1.49 ± 0.19	0.000
**2L by 3L**	0.05 ± 0.06	**0.461**
2L by 3Q	–0.36 ± 0.11	0.002
**2Q by 3L**	0.16 ± 0.11	**0.143**
2Q by 3Q	–1.46 ± 0.19	0.000

a Subsequently, following the proposed statistical analysis, the terms for which the null hypothesis was not rejected (*p* > 0.05) were removed from the model in order to obtain an optimized version. Accordingly, the quadratic effect (Q) of the gap between electrodes (GP), as well as the interaction effects 1L by 2Q, 1Q by 2Q, 1L by 3Q, 1Q by 3L, 2L by 3L, and 2Q by 3L, were removed.

The 1L by 3L interaction effect, which as considered as not significant (or marginally significant) in the initial assessed model, remained within the adopted significance (α = 0,05) threshold in the optimized model, when the other effects were removed. Therefore, the regression coefficients of the optimized model are presented in Table 7, as well as the p-values for each considered effect.

**Table 7:** Optimized Coefficients of the Multiple Linear Regression for the CEF Experiments

variable	coefficient of regression	*p* value
intercept	0.83 ± 0.13	0.000
(1) gap between the electrodes (GP) (L)	–0.33 ± 0.05	0.000
(2) water cut (WC) (L)	0.42 ± 0.12	0.001
water cut (WC) (Q)	1.84 ± 0.16	0.000
(3) surfactant concentration (SC) (L)	–0.31 ± 0.05	0.000
surfactant concentration (SC) (Q)	1.98 ± 0.17	0.000
1L by 2L	0.22 ± 0.06	0.001
1Q by 2L	–0.63 ± 0.11	0.000
1L by 3L	0.16 ± 0.06	0.018
1Q by 3Q	–1.33 ± 0.11	0.000
2L by 3Q	–0.36 ± 0.11	0.002
2Q by 3Q	–1.46 ± 0.19	0.000

Considering the carried-out analysis, the optimized model achieved an *R* ^2^ score of 0.7553, which indicates that approximately 76% of the variability in the response was captured by the new model. When compared with the *R* ^2^ score obtained for the overparametrized model (0.7677), the optimized model showed only a slight reduction in explanatory capacity. Therefore, this small difference indicates that the removed effects had a limited impact on model accuracy, supporting their exclusion from the final formulation.

Although the obtained level of agreement (∼76%) reflects reasonable predictive capability, it also suggests that additional nonlinearities or unaccounted mechanisms may have contributed to the observed variability. Overall, this behavior is consistent with the proposed experimental design, since factors such as local droplet distribution, local variations in the formation of the conductive pathway, and spatial heterogeneities in the emulsion, were not explicitly assessed through the proposed experimental configuration.

Moreover, for better comparing the obtained results, Figure 7 shows the comparison between predicted and observed CEF values, demonstrating satisfactory agreement across the investigated range.

**Figure 7:**
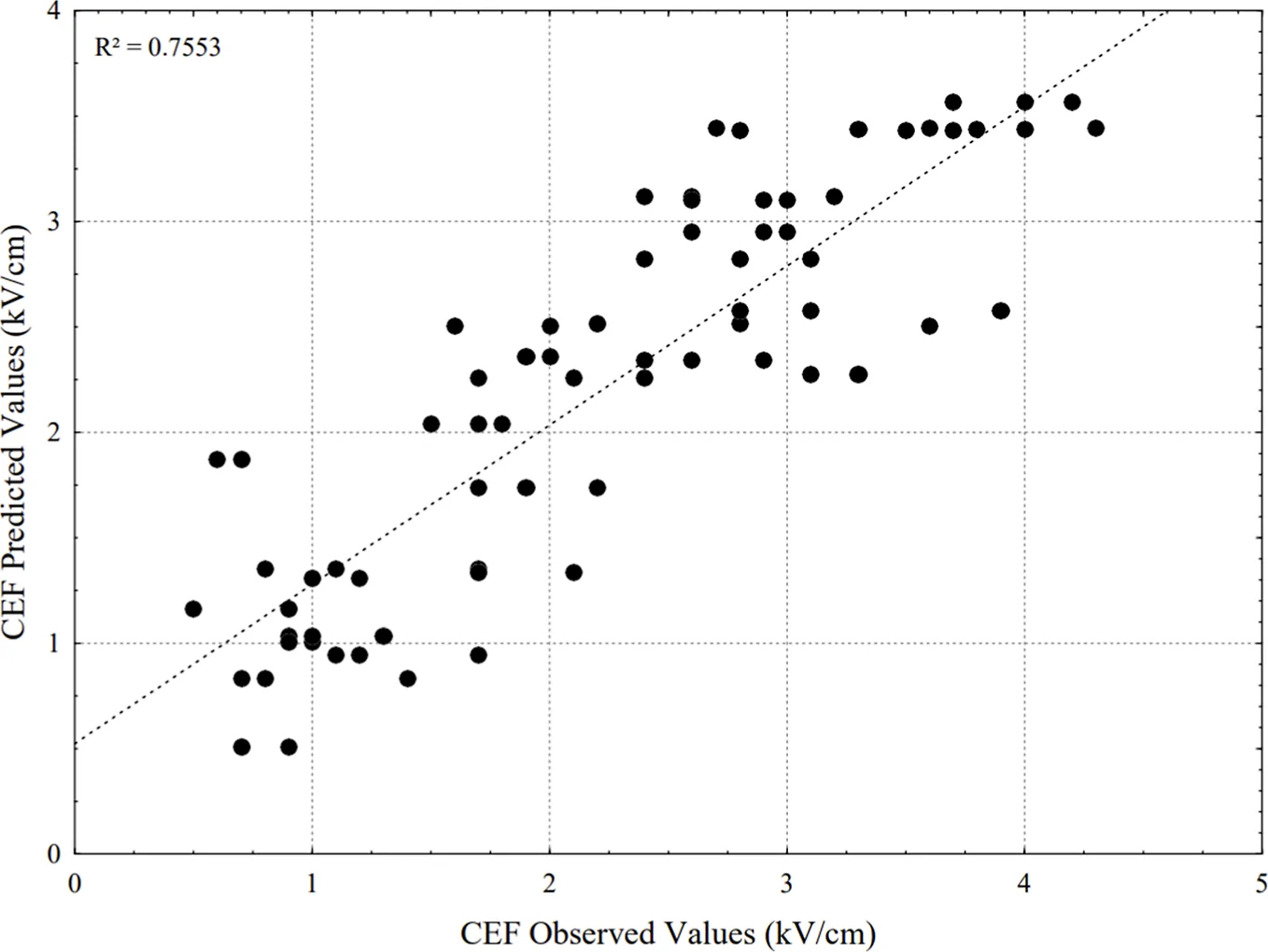
CEF Observed vs CEF Predicted values to evaluate the experiments.

Following the analysis of the regression coefficients presented in Table 7 (and also in Table 6), it indicates that electrode spacing applied a strong influence on the response. The linear term associated with electrode gap displayed a negative coefficient, suggesting a decrease in CEF with increasing spacing within the fitted model. This apparent contradiction with the expected physical trend highlights the importance of considering quadratic effects and interactions, as previously discussed in the literature.^63^ Therefore, this behavior is explained by considering the structure of the data set used in *Statistica* software, in which the experimental scenarios were represented by coded values (−1, 0, 1).

In other words, the regression model obtained from the statistical analysis was fitted using coded variables, rather than the original experimental values. Therefore, the coefficients presented in Tables 6 and 7 describe the response behavior in the coded domain and does not represent coefficients expressed in terms of the real values of the considered variables. In this context, the negative coefficient associated with the linear term of electrode gap reflects the trend estimated within the coded formulation and does not necessarily imply a direct physical reduction in CEF with increasing electrode spacing. In this context, to enable obtaining predictions by using experimental conditions, it is possible to convert the coded model into its real-variable form, as presented in eq 2:

2 $$CEF=0.83-0.33\left(\frac{GP-200}{100}\right)+0.42\left(\frac{WC-10}{5}\right)+1.84{\left(\frac{WC-10}{5}\right)}^{2}-0.31\left(\frac{SC-1}{0.5}\right)+1.98{\left(\frac{SC-1}{0.5}\right)}^{2}+0.22\left(\frac{GP-200}{100}\right)\left(\frac{WC-10}{5}\right)-0.63{\left(\frac{GP-200}{100}\right)}^{2}\left(\frac{WC-10}{5}\right)+0.16\left(\frac{GP-200}{100}\right)\left(\frac{SC-1}{0.5}\right)-1.33{\left(\frac{GP-200}{100}\right)}^{2}{\left(\frac{SC-1}{0.5}\right)}^{2}-0.36\left(\frac{WC-10}{5}\right){\left(\frac{SC-1}{0.5}\right)}^{2}-1.46{\left(\frac{WC-10}{5}\right)}^{2}{\left(\frac{SC-1}{0.5}\right)}^{2}$$

For completeness, the equation obtained after converting the coded model to real variables is explicitly presented in eq 3:

3 $$CEF=0.15\left(GP\right)+2.41\left(WC\right)+66.53\left(SC\right)-0.0004{\left(GP\right)}^{2}-0.16{\left(WC\right)}^{2}-33.88{\left(SC\right)}^{2}+0.01\left(GP\right)\left(WC\right)-0.42\left(GP\right)\left(SC\right)-8.80\left(WC\right)\left(SC\right)+0.21\left(GP\right){\left(WC\right)}^{2}+4.40\left(WC\right){\left(SC\right)}^{2}+0.47\left(SC\right){\left(WC\right)}^{2}-0.00001{\left(GP\right)}^{2}\left(WC\right)+0.001{\left(GP\right)}^{2}\left(SC\right)-0.0005{\left(GP\right)}^{2}{\left(SC\right)}^{2}-0.23{\left(SC\right)}^{2}{\left(WC\right)}^{2}$$

Additionally, from a mechanistic perspective, electrode spacing directly affects electric field distribution and energy input, thereby influencing droplet polarization and coalescence behavior, as reported by Less et al.^62^ Regarding the water cut, it exhibited a positive effect on the critical electric field, which is attributed to reduced interdroplet distances at higher dispersed phase concentrations. As observed in the micrographs (Figure 3) and stability analysis (Figure 4), increased water content enhances emulsion stability and increases the energy required to disrupt interfacial films. The presence of a quadratic contribution associated with water cut is consistent with findings reported by Suemar et al.^64^

The surfactant concentration presented a negative linear effect, which is attributed to increased ionic conductivity arising from the presence of sodium ions associated with AOT, thereby facilitating current flow. However, the positive quadratic contribution indicates that higher surfactant levels enhance interfacial stability through reduction of interfacial tension, increased interfacial elasticity, and strengthening of electrostatic repulsion within the electrical double layer, consistent with established interpretations.^65^

Evaluation of interaction terms revealed that combinations involving electrode spacing, particularly interactions between linear gap and other variables, presented significant influence on the response. These results emphasize that electrode spacing plays a dominant role when coupled with compositional variables, reflecting its direct relationship with electric field intensity and energy distribution within the electrocoalescence cell. Certain interaction terms indicated that increases in water cut combined with changes in electrode spacing modify system conductivity and polarization behavior, thereby affecting the voltage required for destabilization.

Overall, the analysis demonstrates that electrode spacing constitutes a critical parameter governing electrocoalescence performance, particularly when considered in combination with water content and surfactant concentration, highlighting the complex interplay between geometric and physicochemical factors in determining the critical electric field.

## 4 Conclusions

The present study introduced and experimentally validated an alternative criterion for determining the critical electric field (CEF) that differs from conventional definitions widely reported in the literature. The combined use of real-time microscopic observation and electrical current monitoring provided a physically consistent framework for identifying the transition associated with electrocoalescence. The results demonstrated that droplet chain formation represents primarily a manifestation of polarization rather than a definitive indicator of the critical condition. Instead, the abrupt increase in current was associated with progressive droplet growth and the establishment of conductive pathways within the emulsion. These findings support a refined interpretation of the CEF as the field intensity required to promote sufficient droplet alignment and coalescence to enable enhanced current transport, rather than solely the initiation of droplet interaction.

The systematic analysis of 81 experimental conditions revealed that water cut exerted the strongest influence on the critical electric field, reflecting its dominant role in controlling droplet proximity and system conductivity. The observed quadratic dependence confirms its contribution to the nonlinear behavior frequently reported in electrocoalescence systems. Surfactant concentration exhibited a dual effect, in which increased ionic conductivity associated with AOT reduced the critical electric field despite enhanced interfacial stabilization. Electrode spacing influenced the response through its direct relationship with electric field distribution and energy input. Collectively, these results provide new experimental evidence that clarifies the physical interpretation of the critical electric field and highlights the importance of integrating electrical and microstructural analyses.

A limitation of the present study lies in the use of a model water-in-oil system with simplified composition relative to crude oil emulsions, which exhibit additional complexity due to natural surfactants, asphaltenes, resins, and broader physicochemical variability. In addition, the experiments were performed under controlled laboratory conditions using direct current fields and small electrode gaps, conditions that do not necessarily represent the hydrodynamic and electrical environments of industrial separators. Future work should extend the proposed methodology to other water-in-oil systems, including crude oil emulsions with different compositions and interfacial properties, in order to evaluate the generality of the interpretation of the critical electric field.

## Data Availability

All provided information present in this manuscript is properly referred to through the text. There is no further data or code available.
